# Antimicrobial Bilayer Film Based on Chitosan/Electrospun Zein Fiber Loaded with Jaboticaba Peel Extract for Food Packaging Applications

**DOI:** 10.3390/polym14245457

**Published:** 2022-12-13

**Authors:** Luisa Bataglin Avila, Diana Pinto, Luis F. O. Silva, Bruna Silva de Farias, Caroline Costa Moraes, Gabriela Silveira Da Rosa, Guilherme Luiz Dotto

**Affiliations:** 1 Research Group on Adsorptive and Catalytic Process Engineering (ENGEPAC), Federal University of Santa Maria, Roraima Avenue, Santa Maria 97105-900, Rio Grande do Sul, Brazil; 2 Department of Civil and Environmental, Universidad De La Costa, Calle 58 # 55–66, Barranquilla 080002, Colombia; 3 School of Chemistry and Food, Federal University of Rio Grande (FURG), Itália Avenue, Rio Grande 96203-900, Rio Grande do Sul, Brazil; 4 Graduate Program in Materials Science and Engineering, Federal University of Pampa (UNIPAMPA), Maria Anunciação Gomes Godoy Avenue, Bagé 96413-172, Rio Grande do Sul, Brazil; 5 Chemical Engineering, Federal University of Pampa (UNIPAMPA), Maria Anunciação Gomes Godoy Avenue, Bagé 96413-172, Rio Grande do Sul, Brazil

**Keywords:** biopolymer, bioactive compounds, ecofriendly material

## Abstract

This work focused on developing an active bilayer film based on natural extract. Thus, the jaboticaba peel extract (JPE) was produced and characterized and showed promising application as a natural additive in biopolymeric materials. The zein fiber and bilayer films were produced using a chitosan film (casting) and zein fiber (electrospinning), with and without JPE. All samples were evaluated according to thickness, solubility in water, water vapor permeability, and main diameter, and for these, zein fiber, chitosan/zein fiber, and chitosan/zein fiber + 3% JPE showed values of 0.19, 0.51, and 0.50 mm, 36.50, 12.96, and 27.38%, 4.48 × 10^−9^, 1.6 × 10^−10^, and 1.58 × 10^−10^ (g m^−1^ Pa^−1^ s^−1^), and 6.094, 4.685, and 3.620 μm, respectively. These results showed that the addition of a second layer improved the barrier properties of the material when compared to the monolayer zein fiber. The thermal stability analysis proved that the addition of JPE also improved this parameter and the interactions between the components of the zein fiber and bilayer films; additionally, the effective presence of JPE was shown through FTIR spectra. In the end, the active potential of the material was confirmed by antimicrobial analysis since the bilayer film with JPE showed inhibition halos against *E. coli* and *S. aureus*.

## 1. Introduction

Packaging materials are key to ensuring food quality and safety during different commercialization stages, such as storage and distribution. Most of these materials are produced from nonrenewable-based compounds, owing especially to their good properties and low cost [1,2,3]. However, the indiscriminate use of packaging derived from fossil fuel sources has caused serious and irreversible damage to the environment [4,5,6]. Therefore, to minimize the negative impacts promoted by conventional packaging, researchers have paid increasing attention to alternatives that can substitute them, such as the use of biopolymers [2,4,7,8]. Among the various biopolymers available in nature, chitosan and zein stand out. The first is the second most abundant polymer after cellulose. Besides that, chitosan is known for its important properties, such as antimicrobial and antioxidant activity, biodegradability, and nontoxicity [9,10]. The second is the product of the wet milling of corn grain and is the main protein of this grain, representing approximately 80%. Despite being a protein, zein has low nutritional value and is not indicated for human consumption. In line with this, its properties, such as high hydrophobicity, a high oxygen barrier, and thermal resistance make zein a great biopolymer option for food-packaging-material application [11,12].

On the other hand, although the packaging produced with biopolymers is an ecofriendly material, it still faces obstacles that hinder its competitiveness against conventional materials. Such obstacles are mainly related to the barrier and mechanical properties that are inferior to synthetic packaging [4,13]. Strategies such as the use of materials to reinforce the biopolymer packaging films are reported in the literature, such as the study developed by Oyeoka et al. [14], which describes the use of cellulose nanocrystals extracted from water hyacinth to reinforce PVA-gelatin films.

An important strategy to improve the properties of bio-based packaging is the development of materials with two or more layers. In this type of packaging system, it is possible to add different materials to the same packaging formulation, which makes it possible to optimize the properties of the final product by adding the best characteristics of each biopolymer used [1,13]. However, this structure requires further studies and advances, and the possibility of the controlled release of active compounds is one of its challenges. In this sense, the electrospinning technique allied to the multilayer system can promote this characteristic. The main advantage of using electrospun fibers relates to the production method, which does not use high temperatures, and to the inherent characteristics of the fibers, such as high surface area, good malleability, and being lightweight [15,16,17,18]. Thus, many researchers have made efforts to develop the bilayer system. For example, Ebrahimzadeh et al. [18] developed a bilayer film consisting of the electrospun chitosan-poly (vinyl alcohol) incorporated with essential oils and laminated onto a chitosan film. Martins et al. [19] described the development of an active bilayer based on electrospun ethyl cellulose and a microfibrillated cellulose film incorporated with cinnamaldehyde. Nilsuwan et al. [20] reported a study about the bilayer films based on poly (lactic acid) and fish gelatin with added epigallocatechin gallate. 

Still, regarding additives in food packaging, consumer demand for minimally processed products has driven industries to invest in technologies that allow for the use of natural additives over synthetic additives. Therefore, agroindustrial residues and byproducts are highlighted in the search for sources rich in phytochemicals [21,22]. Once added to the polymer matrix, natural additives can provide the packaging system with antioxidant and antimicrobial properties, thus increasing the shelf life of the packaged food. This type of material is known as active packaging and has received increasing attention over the years. [23,24]. Fabra et al. [25] described a study about active bilayer films based on the electrospinning/electrospraying of different polymer matrices that are incorporated with alpha-tocopherol and deposited into a thermoplastic gluten film. Estevez-Areco et al. [26] reported the development of bilayer films using cassava starch and PLA fibers containing rosemary extract with antimicrobial properties. In line with environmental and food safety concerns, jaboticaba peels (JPs) are an interesting alternative as a source of phenolic compounds. The use of JP makes it possible to add value to a material that would be discarded, and this also confers important properties to food packaging materials, such as antioxidant and antimicrobial properties [27,28]. 

Some works have already been developed and have demonstrated the potential of using JP as a source of bioactive compounds. For example, Avila et al. [29] developed a carrageenan-based film with jaboticaba peel extract (JPE) obtained by microwave extraction. Avila et al. [30] also used the JPE, incorporating it into the zein fibers obtained using the electrospinning technique. On another point, Avila et al. [31] used the JPE to obtain active and intelligent films for food packaging applications. Recently, Valério Filho et al. [32] described the promising recovery of phenolic compounds from agroindustrial residues, including jaboticaba peel, with important properties such as antioxidant, antimicrobial inhibition, and bactericide from ethanolic extracts.

Furthermore, considering environmental concerns and human health, using biopolymers in line with natural additives in a single packaging material can be achieved using a bilayer system. Additionally, despite the promising characteristics of electrospun fibers, they have a limiting factor that makes their application as food packaging difficult. This restriction is related to mechanical properties since, according to Ebrahimzadeh et al. [18], fibers obtained by electrospinning are fragile and need a substrate that can support them. This fact reinforces the relevance of approaches focused on combining electrospun fibers with biopolymers films in a bilayer system. Thus, the association of electrospun fibers in a bilayer system can also contribute to obtaining a final packaging product with enhanced characteristics and unique properties, such as sustained bioactive release. 

In this context, the objective of this work was to develop an antimicrobial bilayer system based on two biopolymers: chitosan and zein, and agroindustrial waste, JP, as a source of bioactive compounds. Therefore, a casting technique produced the chitosan layer, and was configured as an outer layer acting as a substrate for the inner layer. On the other hand, zein layers were produced by the electrospinning technique. Therefore, they were chosen as inner layers to be in contact with the packaged food since JPE (the active compound) was added to this layer.

## 2. Materials and Methods

### 2.1. Reagents

Chitosan (Êxodo Científica, Sumaré, SP, Brazil), glycerol, 2,2-diphenyl-1-picrylhydrazyl (DPPH), Folin Ciocalteu’s phenol, anhydrous sodium carbonate, zein (Sigma Aldrich, St. Louis, MO, USA), and acetic acid were of analytical grade. Water (Milli-Q), acetonitrile, and standards of cyanidin-3-glucoside (Cn-3-Glu), caffeic acid, quercetin, Kaempferol, trans-ferulic acid, trans-cinnamic acid, p-coumaric acid, caffeic acid, and gallic acid were of HPLC grade. For the antimicrobial test, Nutrient, Müller-Hinton broth was used. All reagents were purchased from Sigma Aldrich (St. Louis, MO, USA). The bacteria strain used in the antimicrobial test were *Escherichia coli* K12 (ATCC 10798) and *Staphylococcus aureus* (ATCC 12598), gained from Cederlane (Burlington, CA, USA).

### 2.2. Sample Preparation

The jaboticaba fruits (*Plinia cauliflora*) were acquired from a private farm in Santa Maria (−29.88926, −53.87125), Rio Grande do Sul, Brazil. Preparation and sanitization followed the methodology described by [30].

### 2.3. Extraction Procedures

The extraction step was performed using the maceration technique in a Dubnoff metabolic bath (SOLABSL-157/30, Piracicaba, Brazil) at a ratio of 1:100 (particle/solvent) for 2 h at 88 °C, according to the proposed method by Valério Filho et al. [32]. The solvent used was aqueous ethanol (40%), and the choice of solvent was based on its low toxicity for use with the extract as an additive for food packaging. After extraction, the samples were vacuum filtered. Then, the extracts used in the formulation of the fibers were frozen and freeze-dried, while the extracts for analysis were immediately used.

### 2.4. Extract Characterization

The extracts were characterized according to total phenolic content (TPC), total anthocyanin (TA), antioxidant activity (AA), and phenolic compounds using high-performance liquid chromatography (HPLC). The methodologies used in each analysis are described below; all were conducted in triplicate.

#### 2.4.1. Total Phenolic Content (TPC)

The TPC of the extracts was evaluated according to the methodology proposed by Singleton and Rossi [33]. Thus, the TPC was measured using a gallic acid standard curve at concentrations ranging from 0–400 mg L^−1^. The results were expressed as mg of gallic acid equivalent (GAE) per gram of dry mass.

#### 2.4.2. Total Anthocyanin (TA)

The TA present in the extracts was evaluated using a spectrophotometer method, as described by Avila et al. [30], at 520 nm, concerning the absorbance of Cn-3-Glu: the main anthocyanin in JP. The TA was measured using a standard curve of Cn-3-Glu at concentrations ranging from 5–100 mg L^−1^, and the results were expressed as mg of Cn-3-Glu equivalent per 100 g of dry mass.

#### 2.4.3. Antioxidant Activity (AA)

The AA was evaluated using the methodology proposed by Brand-Williams, Cuvelier, and Berset [34], and the results were expressed as the percentage of free radicals scavenged by DPPH radicals.

#### 2.4.4. High-Performance Liquid Chromatography (HPLC) Analysis

HPLC analysis was used to identify and quantify the compounds present in the extracts. Then, HPLC analysis was carried out using an Agilent 1260 Infinity Series High-Performance Liquid Chromatography (HPLC) instrument (Santa Clara, CA, USA) equipped with a variable wavelength (VWD) detector. A gradient method was used, and the separation was performed at 30 °C using an Eclipse Plus C18 reverse phase LC column (4.6 × 150 mm, 5 µm) (Supelco, Bellefonte, PA, USA). The injection volume and mobile phase flow rate was set at 20 µL and 1 mL min^−1^, respectively. The sample preparation and analysis procedures followed the method previously studied and described by Valério-Filho et al. [32], analyzing at two wavelengths: 280 nm and 520 nm. The identification of the compounds was performed by comparing their retention times to pure Sigma-Aldrich^®^ standards (Steineheim, Germany), and they were quantified using the standard curves of compounds known to be present in JP.

### 2.5. Bilayer and Monolayer Film Formation

**Figure 1:**
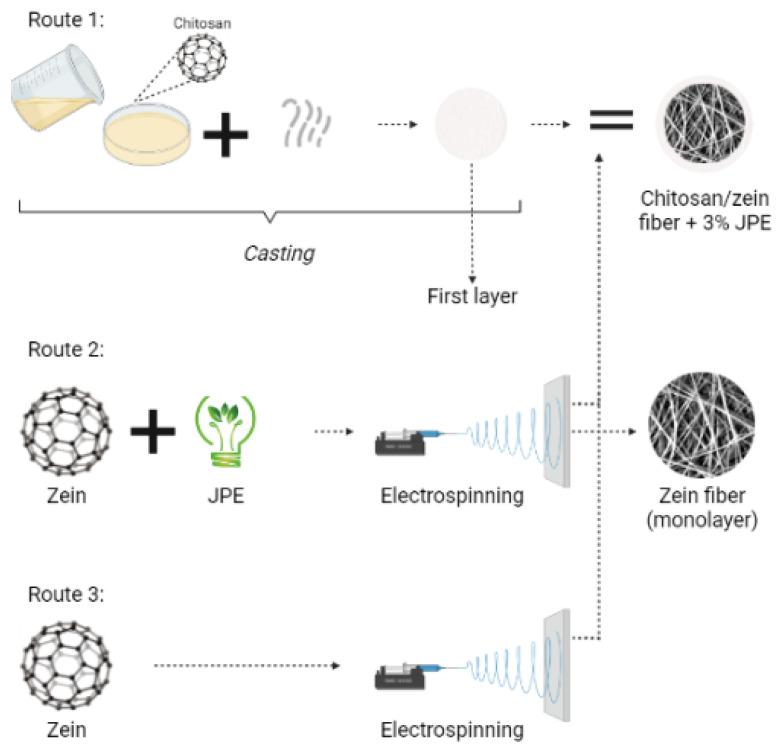
Scheme of the experimental procedure to obtain the bilayer and monolayer films.

The development of bilayer films, as well as obtaining the zein fibers, was carried out as described in Figure 1.

#### 2.5.1. Chitosan Film Formation

With some modifications, chitosan films were prepared by a casting technique with proportions and conditions described by Soares et al. [35]. In this sense, the chitosan-based films were obtained from 1 g of biopolymer dissolved in 70 mL of acetic acid (1%); this step was carried out overnight. Then, 0.3 g of the plasticizer (glycerol) was added to the biopolymer solution and homogenized in a mechanical shaker with light agitation for 10 min. Then, the samples were poured into polystyrene Petri dishes (150 mm diameter). Next, the drying step was carried out in a convective dryer at 40 °C for 24 h. Lastly, the biopolymer films were withdrawn from the plates and maintained at 50% relative humidity at room temperature until the next procedure to obtain multilayer films.

#### 2.5.2. Zein Solutions Preparation

The zein solutions were prepared according to Antunes et al. [36]. The methodology consists of dissolving 0.9 g of biopolymer in 3 mL of ethanol 70% (*v*/*v*) and stirring at room temperature for 1 h. A zein solution containing 3% (*w*/*v*) of lyophilized JPE was also prepared using the same method. The difference consisted only in previously dissolving the lyophilized JPE in ethanol 70% (*v*/*v*) and after adding the zein polymer and stirring.

#### 2.5.3. Apparent Viscosity (AV) and Electrical Conductivity (EC) of the Zein Solutions

AV was performed using a rheometer (Brookfield DV-II+Pro, Devon-Berwyn, PA, USA) coupled with a No. 62 spindle. All measurements were conducted at a 100 rpm speed and a temperature of 25 ± 2 °C. The EC analyses were carried out using a conductivity meter (LF1, handylab LF1, Schott Glaswerke Mainz, Mainz, Germany), made at a temperature of 23 ± 2 °C and are expressed as μs cm^−1^. All tests highlighted in this section were carried out in duplicate.

#### 2.5.4. Electrospinning Procedures

The electrospinning technique produced zein fibers under the conditions and parameters established by Antunes et al. [36]. Firstly, the collector was covered with aluminum foil for the monolayer film; after, the collector was covered with chitosan-based film for the multilayer system. The distance between the collector and the needle was fixed for all samples at 10 cm. The process was carried out at a temperature of 23 ± 2 °C, controlled by an air conditioner and a dehumidifier with a relative humidity of 45 ± 2%. 

### 2.6. Characterization of the Zein Fiber and Bilayer Films

#### 2.6.1. Thickness

The zein fiber and bilayer films were evaluated according to their thickness, and the analysis was performed using a digital micrometer (Insize-IP65, São Paulo, Brazil). The accuracy of the micrometer was 0.001 mm, and the analyses were measured at 10 random locations on the zein fiber and bilayer films.

#### 2.6.2. Solubility in Water (SW)

The SW of the zein fiber and bilayer films was evaluated according to the methodology proposed by Gontard and Guilbert [37]. Thus, the samples were cut into 2 cm diameter disks, dried at 105 °C for 24 h, and weighed to determine the initial dried mass. The next step consisted of placing the dried samples in recipients with 50 mL of distilled water. Afterward, the sets were submitted to constant agitation (100 rpm) in an incubator shaker (Solab, SL 223, Brazil) at room temperature for 24 h. After this period, the undissolved samples were filtered and dried at 105 °C for 24 h to define their final dry mass. Finally, the *SW* (%) was quantified by Equation (1).

(1) $$SW=\frac{m_{i}-m_{f}}{m_{i}}100\%$$

where, $m_{i}$ is the initial dry mass (g) and $m_{f}$ is the final dry mass (g).

#### 2.6.3. Water Vapor Permeability (WVP)

The WVP of the zein fiber and bilayer films was performed according to the standard ASTM E 96/E 96M-16 [38]. Thus, the samples were sealed in permeation cells containing anhydrous calcium chloride and stored in desiccators with a relative humidity of 50% at room temperature. The permeation cells were weighed on the first day and after seven days to determine the calcium chloride mass gain. The results were quantified using Equation (2) and expressed as g m^−1^ Pa^−1^ s^−1^.

(2) $$WVP=\frac{W_{a}}{t}\frac{l}{A\cdot \Delta P}$$

where, $W_{a}$ is the amount of absorbed water (g), *t* is the total analysis time (s), *l* is the average film thickness (m), *A* is the area of the exposed film surface (m^2^), and Δ*P* is the partial vapor pressure difference across the samples (Pa).

#### 2.6.4. Morphology and Mean Diameter of Zein Fibers

The morphology of the multilayer films, as well as the electrospun zein fiber, was evaluated using two methods: Scanning Electron Microscope (SEM) (Jeol, JSM-6060LVAkishima, Tokyo, Japan) and Atomic Force Microscopy (AFM). The samples were evaluated for the AFM analysis, and the electrospun fibers mean diameter was defined according to the micrographs based on 60 random fibers. The maps were recorded on a Park NX10 microscope (Park Systems, Suwon, Korea) equipped with SmartScan version 1.0.RTM11a. The measurements were conducted using PPP-EFM (Nanosensors, Neuchâtel, Switzerland).

#### 2.6.5. Thermal Stability

The thermal stability of the zein fiber and the bilayer films was evaluated using a thermogravimetric instrument (Shimadzu, TGA 50, Kyoto, Japan). The samples, approximately 5 mg, were heated in platinum capsules at 30–600 °C at a heating rate of 10 °C min^−1^ with a nitrogen flow of 50 mL min^−1^. 

#### 2.6.6. FTIR Analysis

The Fourier transform infrared (FTIR) analyses were used to identify the functional groups in the zein fiber and bilayer films and the interaction between the extract and biopolymer. Therefore, a spectrometer (Shimadzu, Prestige 21, Nakagyo-ku, Kyoto, Japan) was used from 500 cm^−1^ to 4000 cm^−1^.

#### 2.6.7. Microbial Inhibition Potential (MIP)

The zein fiber and bilayer films, with and without JPE, were evaluated for microbial inhibition potential against two microorganisms, *Escherichia coli* (*E. coli*) and *Staphylococcus aureus* (*S. aureus*). The analyses were performed according to the disk-diffusion method [39]. Previously, bacterial cultures were prepared in nutrient broth (HIMEDIA) and incubated at 35 °C for 24 h in a bacteriological incubator (SOLAB, SL 101). After incubation, the microorganism concentration was adjusted to 10^4^ CFU/mL. This inoculum was evenly spread on sterilized Petri dishes containing Mueller-Hinton agar. Next, the samples, cut into disks and previously sterilized in ultraviolet light, were placed in contact with the contaminated Mueller-Hinton agar, and the system was incubated at 37 °C for 24 h. After this period, the inhibition halos were visually verified and measured using a digital pachymeter.

### 2.7. Statistical Analyssis

Experimental data were analyzed by Statistica^®^ software, version 10.0 (SAS Institute, Cary, NC, USA). The mean comparisons were carried out by Tukey test, and *t*-tests were applied for determining the significant differences at 95% significance level.

## 3. Results

### 3.1. Extract Features

The results of the total phenolic compounds, anthocyanin, and antioxidant activity were, respectively, 98.88 ± 1.79 mg_GAE_ g^−1^, 1075 ± 2.97 mg_cn-3-glu_ 100 g^−1^, and 89.52 ± 0.63%. These findings prove the excellent potential to use JP as a source for bioactive compounds. Thus, the values mentioned above agree with those found in the literature. For example, Mattos et al. [40] evaluated the total phenolic compounds of the hydroethanolic extract of JP under different solvent concentrations, extraction temperatures, and solid-to-liquid ratios. As a result, the authors observed that using 50% ethanol (*v*/*v*), an extraction temperature of 57 °C, and a solid-to-liquid ratio of 1:10, it was possible to achieve a value of 106 mg_GAE_ g^−1^. This result is very close to our work. Besides, it is important to highlight that, although the authors achieved this value using a lower temperature than the present study, the reported extract is more concentrated. In their study, Avila et al. [31] used acidified water as a solvent to recover bioactive compounds from JP. As a result, the authors found a total phenolic compound value of 199.34 mg_GAE_ g^−1^. This difference can be attributed to the acidic solvent and the fact that the raw material obtained was from a different harvest period. Paludo et al. [41] reported differences in the composition of jaboticaba fruits in different harvesting years and attributed them to the climate conditions since the fruits are submitted to different temperatures and exposures of UV light.

As well as the total phenolic compounds, the total anthocyanin content of JPE also showed an interesting result. This value is higher than Barros et al. [42], which evaluated the influence of acid type in the extraction of bioactive compounds and achieved a maximum total anthocyanin content of 340 ± 0.1 mg 100 g^−1^. Along with phenolic compounds, anthocyanins play important functions, with highlighted antimicrobial and antioxidant potential. In this sense, the result of the DPPH reagent scavenging obtained in the present work corroborates this statement.

This value is very promising compared to other works that used JP as a source of bioactive compounds. For example, Avila et al. [29] describe the study of microwave-assisted extraction conditions to recover bioactive compounds from JP and reported values ranging from 90.9 ± 0.6% to 95.4 ± 0.2%. In addition, Pitz et al. [43] report a similar value for DPPH reagent scavenging: 91.01 ± 0.42% from JPE using microwave-assisted extraction and acidified ethanol 50% solution as the solvent.

**Figure 2:**
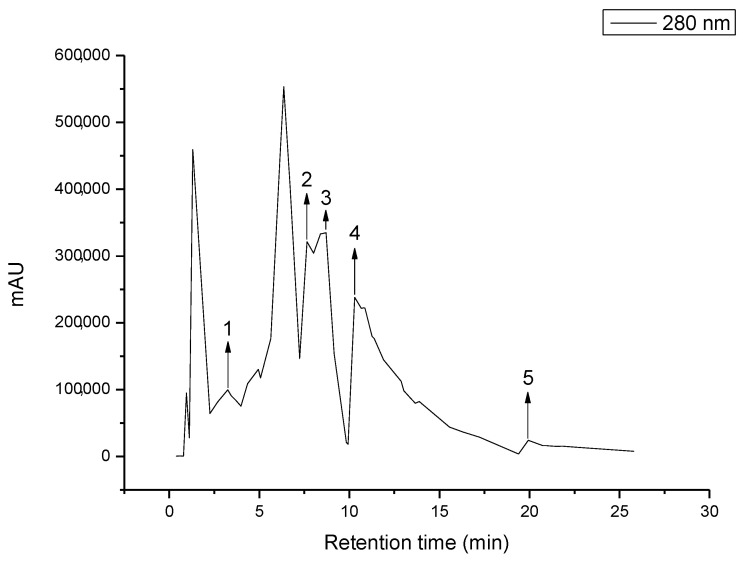

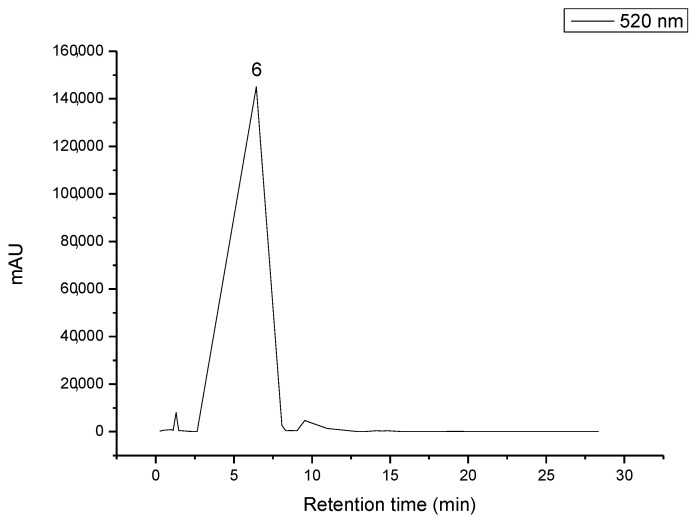
HPLC chromatograms of JPE. 1. Gallic acid, 2. Caffeic acid, 3. p-Coumaric acid, 4. trans-Ferulic acid, 5. Kaempferol, 6. Cyanidin-3-glucoside.

Thus, the HPLC analysis was carried out to better understand the composition of JPE and which compounds are possibly responsible for its antimicrobial action. The results and chromatograms are presented in Table 1 and Figure 2, respectively.

The results above corroborate the results of the total phenolic compounds and anthocyanins. As expected, the major content present in this extract corresponds to Cn-3-Glu, with 75.42% of the content of the total compounds identified. Leite-Legatti et al. [44] already reported this fact in their study. The highest concentration for JP was obtained for cyanidin-3-glucoside, and they related a value of 1514.82 ± 45.51 mg 100 g^−1^ for the extract using a solution of methanol/water/acetic acid as a solvent. In addition, other important compounds have been identified in the extract, but at low concentrations, such as gallic acid, caffeic acid, *p*-Coumaric acid, *Trans*-Ferulic acid, and kaempferol. Inada et al. [45] studied the phenolic profile of JPE and also reported the presence of Cn-3-Glu (1261 ± 18 mg 100 g^−1^) and gallic acid (21 ± 1 mg 100 g^−1^). 

Although the other compounds identified in the present study presented much lower concentrations than cyanidin-3-glycoside, they still play an important role in antioxidant and antimicrobial functioning. This trend is especially due to the synergistic effects of the constituents of the extract. Some authors have already described that combinations of bioactive compounds present more benefits than the individual component, as the individual compound can be altered in the presence of others. With this, there is a potentiation of important properties such as antimicrobial [46,47,48].

Rodriguez-Pérez et al. [49] evaluated the antimicrobial potential of the phenolic compounds isolated from cranberries, such as quercetin, kaempferol, and caffeic acid, against *E. coli*. They compared it with the antimicrobial effect of cranberry extract. The authors observed that the combined compounds in the extract had a better effect than the isolated compounds. Among the isolated compounds also identified in JP are quercetin, kaempferol, and caffeic acid. Therefore, based on extract characterization, the results suggest various applications, such as pharmaceutical, cosmetology, and functional additives in food and packaging where antioxidant and antimicrobial properties are desired.

### 3.2. AV and EC of the Zein Solutions

AV and EC are two important factors in polymer solutions for the electrospinning process. This behavior is due to these parameters being associated with fiber formation capacity and morphology [50,51,52]. In this sense, the zein solutions’ AV and EC were performed, and the results are presented in Table 2.

The results presented above suggest that the addition of JPE into the zein matrix significantly increased both of the parameters evaluated. The same behavior was reported by Avila et al. [30], who incorporated JPE into a zein solution to form a monolayer zein fiber. This fact can be associated with the extract composition, which is rich in phenolic acids [53]. 

Regarding the AV of the polymeric solution, in their study on the importance of the viscosity of polymeric solutions in electrospinning, Tiwari et al. [54] stated that this parameter must be high enough to overcome surface tension. In this sense, the results in Table 2 are consonant with those proposed in the literature. 

Krumreich et al. [17] evaluated 20, 25, and 30% (*w*/*v*) zein solutions incorporated with 0, 15, and 30% (*w*/*w*) avocado oil and found values ranging from 0.0387 Pa s to 0.14943 Pa s. The highest value corresponds to a zein solution at 30% (*w*/*v*) incorporated with 30% (*w*/*w*) of avocado oil. Based on this, it is possible to observe a similarity in the behavior of the polymeric solution when incorporating an active compound. In another study, Miri et al. [55] also evaluated the AV of zein solutions at 26% *w*/*v* and reported a value of 0.492 Pa s. Besides, Horus et al. [56] found values very close to those obtained in the present study, ranging from 0.425 to 0.521 Pa s for solutions containing different tomato peel extract concentrations.

As well as the AV, EC is one of the most important factors in a successful electrospun fiber formation. According to Andrady [57], polymeric solutions need the minimum EC to be electrospun. The values obtained were adequate for the electrospinning process and agreed with those proposed in the literature. Antunes et al. [36] evaluated the EC of a zein solution with different concentrations of eucalyptus essential oil and obtained values ranging from 185.0 ± 2.0 to 238.0 ± 1.0 μs cm^−1^. As mentioned before, Avila et al. [30] also developed zein solutions with JPE and reported an EC value of 256.4 ± 1.2 μs cm^−1^ for a zein solution with 3.3% JPE.

### 3.3. Features of the Zein Fiber and Bilayer Films

The zein fiber and bilayer films were evaluated according to their thickness, solubility in water, and water vapor permeability, and the results are presented in Table 3.

The results showed that the thickness ranged from 0.19 ± 0.03 to 0.50 ± 0.05 mm, with a significant increase related to adding a second layer. When comparing the bilayer film without an extract with the bilayer film with added JPE, no significant increase in thickness was observed. The values observed in the present study are very close to those reported by Cai et al. [58] for a polycaprolactone/curcumin-loaded gelatin/polycaprolactone multilayer film, who found a value of 0.581 mm. Martins et al. [19] developed bilayer structures based on microfibrillated cellulose with and without cinnamaldehyde, which was obtained via casting and electrospinning methods, and reported thickness values ranging from 0.081 ± 0.002 to 0.484 ± 0.098 mm.

Concerning the significant thickness changes between the zein fiber and bilayer films, some authors described the same behavior, such as Wang et al. [59]. The latter evaluated the thickness of the individual electrospun dextran layer and the trilayer structure (cast gelatin as outer layers and electrospun dextran as an inner layer), obtaining a significant difference between the same features. Although there is a significant increase in thickness with the addition of the second layer, the same behavior was not observed with the addition of the extract compared to the bilayer films. This fact can be related to the process formation of the membranes since the extract was added to the electrospun zein fiber. Radusin et al. [60] added *Allium ursinum* L. extract to electrospun polylactide fiber and did not observe a significant difference in thickness compared to the control fiber. Figueroa-Lopez et al. [61] developed a multilayer system based on polycaprolactone fibers and gelatin film, adding black pepper oleoresin into the fiber layer and observing a small increase in thickness. 

Solubility can be explained by the affinity between the material and a substance which is an essential parameter in food packaging material applications [62,63]. Based on the solubility response, it is possible to understand the material’s behavior when in the presence of water. This parameter is an important tool in choosing the purpose of the material [64]. In this sense, the solubility results in water showed a significant reduction by adding a chitosan layer when comparing the zein fiber with bilayer film without JPE. On the other hand, the difference between the water solubility of the zein fiber and the bilayer film decreased with the addition of JPE. Although there is no significant difference between the zein fiber and bilayer film with JPE, it is possible to observe a reduction trend of water solubility by adding a second layer. This result highlights one of the main purposes of using the bilayer structure: to improve the characteristics of the final product by using different polymers.

Some authors reported similar behavior, such as Zhang et al. [65], who described the use of zein and chitosan biopolymers to develop a bilayer film using a layer-by-layer technique and showed an improvement in the barrier properties and water resistance. The authors found approximately 34% and 15% values for a chitosan film and a bilayer chitosan/zein film, respectively. Ferreira et al. [66] developed a bilayer film based on chitosan and FucoPol using the casting method. They found a result of 33.6 ± 3.6% for this parameter, which is higher than what we observed in the present study. From another perspective, Martiny et al. [67] developed a monolayer film based on carrageenan, a biopolymer known for its high hydrophilicity, and added olive leaf extract. As a result, the authors observed that the extract did not cause a significant change inthe water solubility of the carrageenan films, as was true in this work.

As well as the water solubility parameter, water vapor permeability is also important to assist in the decision on the final application of the material, as it is directly related to the product’s shelf life [66]. Thus, the bilayer films were evaluated according to this parameter, and the values are shown in Table 3. These results are in agreement with reports in the literature. 

Soares et al. [35] developed a monolayer chitosan film and observed a similar result (to the present work) for a bilayer chitosan/zein film. The authors reported a value of 2.11 ± 0.02 g m^−1^ Pa^−1^ s^1^. On the other hand, the literature showed higher values than those observed here from the point of view of a zein fiber. Altan et al. [68] found values ranging from 2.64 × 10^−4^ to 6.53 × 10^−4^ g m^−1^ Pa^−1^ s^−1^ for WVP in a zein ultrafine fiber encapsulated with carvacrol. Based on this, it is possible to observe an improvement in the barrier property using a structure with two layers. This improvement is more expressive concerning the zein fiber than the chitosan film. This trend can be attributed mainly to the method of obtaining the materials. Electrospun fibers are known for their high porosity and can contribute to the increased permeability of water vapor [18]. Regarding the bilayer films, Zhang et al. [65] developed a similar material, a chitosan/zein bilayer film, but using the layer-by-layer technique and different proportions of polymers. The authors found values of WVP ranging from 1 × 10^−12^ to 2 × 10^−12^ g m^−1^ Pa^−1^ s^−1^.

In general, the WVP showed the same behavior as the solubility in water and was also improved by adding a chitosan layer. This significant reduction is a positive aspect since the aim is to apply this material in food packaging. Andrade et al. [1] compared the WVP of a PLA monolayer film and PLA-PVA-PLA multilayer films and observed that adding layers improved this parameter in all the ratios evaluated. According to Tampau et al. [69], using two or more layers with different biopolymers forming a single package structure is a methodology that has been successfully applied to improve the barrier properties of the materials.

### 3.4. Morphology and Main Diameter of Zein Fibers

**Figure 3:**
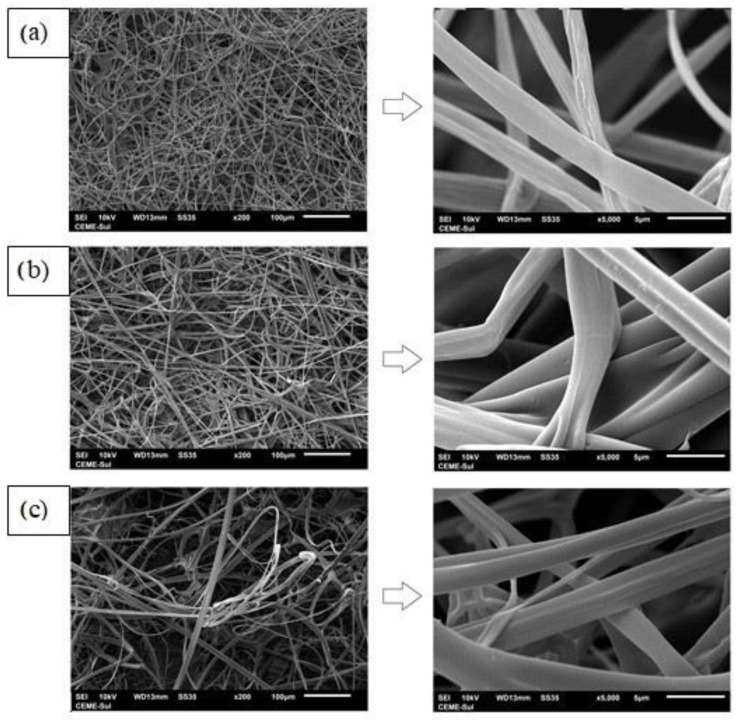
Morphology of zein fiber (**a**), chitosan/zein fiber (**b**) and chitosan/zein fiber + 3% of JPE (**c**). JPE: jaboticaba peel extract.

The morphology of the zein fiber and the bilayer films was evaluated using a scanning electron microscope, and the micrographs are shown in Figure 3.

From Figure 3, it is possible to observe that all the fibers studied present a continuous and homogeneous format. In addition, the presence of beads was not observed. Therefore, this result confirms that the deposition of a zein fiber into the chitosan film surface and the addition of JPE into the zein polymeric matrix did not affect fiber morphology. Quiles-Carrillo et al. [70] reported a similar result when adding gallic acid to PLA fibers, where no variations in their morphology were observed. Avila et al. [30] also added JPE to zein fibers and did not observe a change in the fiber morphology. In addition, it is possible to observe fiber-to-fiber adhesion, which is a positive factor for the application of food packaging, as, according to the literature, good adhesion directly implies good mechanical properties [71,72]. However, due to the complexity of the adhesion effect, which is related to the different configuration of the fibers (cross cylinder and parallel fiber), more and specific studies are needed.

**Figure 4:**
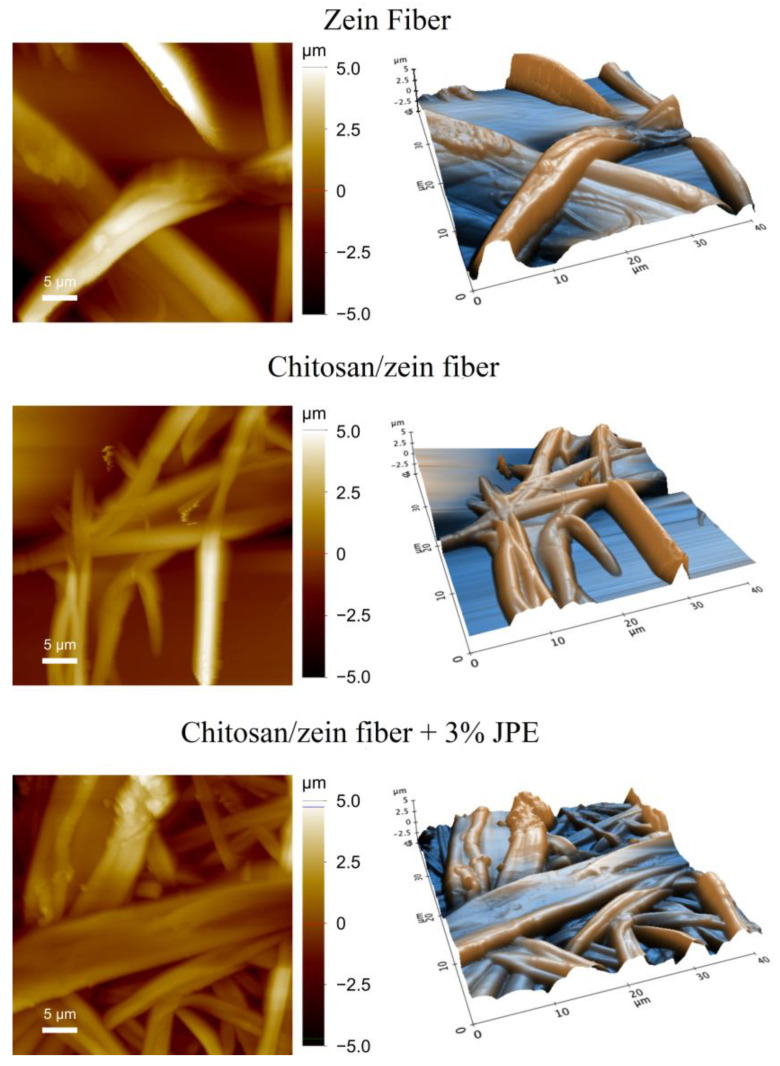
AFM images of zein fiber, chitosan/zein fiber, and chitosan/zein fiber + 3% of JPE.

The morphology of the zein fiber and the bilayer films was also evaluated using AFM analysis. Based on this, it was possible to visualize the changes in the roughness and determine the average diameter. Figure 4 shows the AFM analysis of the samples, and Table 4 presents the obtained results for roughness and mean diameter.

Regarding the results presented above, it is possible to observe a reduction in the roughness of the materials. This reduction is associated with the flattening of the fibers, which is evidenced in Figure 4. In addition, the morphology of the fibers, which are ribbon-shaped (previously suggested by the SEM images), can be confirmed by the AFM analysis. The same format of a zein fiber has already been described in the literature. In their study, Torres-Giner et al. [73] verified that the thinnest fibers seemed to have a tubular shape. However, the ribbon shape can be confirmed in fibers with an intermediate size. Koombhongse et al. [74] suggest that the formation of fibers in the form of a ribbon is favored by a process in which, after the skin is formed, the solvent inside the jet escapes. The action of atmospheric pressure causes the jet of the tube to collapse. As a result, the previously circular cross-section becomes elliptical and then flat.

Koca and Bayramoglu [75] associated high roughness with the high porosity of the material. In this sense, the roughness results mentioned above agree with the WVP results shown earlier. Therefore, from the point of view of food packaging application, the reduction in roughness is considered a positive fact.

On the results presented in Table 4, the average diameter of the fibers was reduced by the addition of JPE in a zein solution. This fact is directly associated with the characteristics of zein solutions, such as AV and EC. Rodriguez-Tobias et al. [52] highlight that the increase in EC affects fiber morphology, resulting in fibers with a smaller average diameter. This behavior can be explained by the greater number of charges around the droplets.

The characterization of the polymeric solutions described above confirms this statement since the addition of JPE into zein solutions caused an increase in both parameters (AV and EC). Some authors have described the development of zein fibers with different additives, as well as different applications. Among the works found in the literature, different average fiber diameters are reported. This variety can be attributed to several factors inherent to the composition of film-forming solutions (AV and EC) and the parameters associated with the process, such as collector distance, flow rate, and applied voltage [76].

Yao et al. [77] developed zein fibers with different concentrations of polymer and different concentrations of solvent (aqueous ethanol). The authors found different values for a diameter that varied to 500 nm for fibers with the presence of beads and 1–6 μm for ribbon-like fibers without beads. Selling et al. developed zein fibers at 30% (*w*/*v*) using aqueous ethanol at 80% (*v*/*v*) and obtained fibers with a mean diameter of 2.1 μm. Based on this, it is possible to observe that the fiber diameter found in the present study is in line with that proposed in the literature. Thus, the results confirm the potential use of this material in several areas, with a special focus on active food packaging, aiming at the controlled release of the active compounds trapped in the fibers.

### 3.5. Thermal Stability

**Figure 5:**
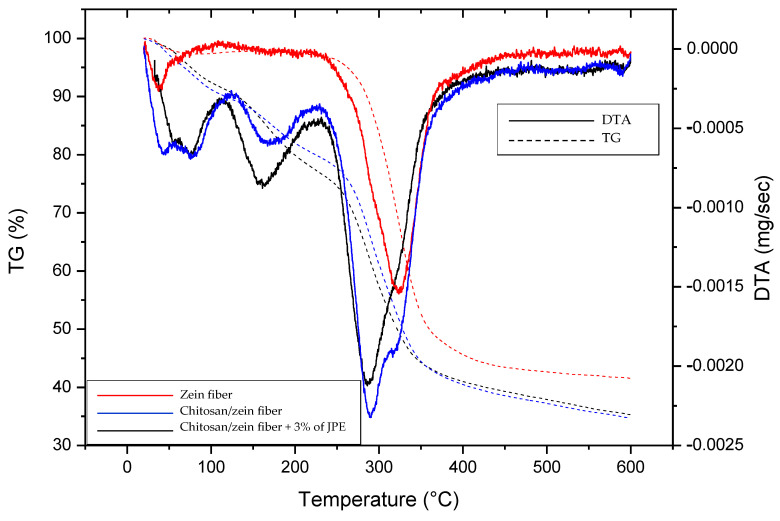
TG and DTA curves of zein fiber, chitosan/zein fiber, and chitosan/zein fiber + 3% of JPE.

Analysis of the thermal stability of the zein fiber and the bilayer films was performed, and the results are shown in Figure 5 through the TG and DTA curves.

The degradation stages show differences between the zein fiber and bilayer films. The first degradation zone for all samples (until approximately 100 °C) can be related to water evaporation. After that, the zein fiber shows just one more stage of degradation from 233–360 °C. The rapid decomposition is reported in the literature as zein polymer structure degradation [65,78]. The bilayer films show a degradation stage at around 150–210 °C, which can be attributed to the decomposition of glycerol and chitosan, and the most pronounced stage was at 250–370 °C. According to Marroquin et al. [79] and Zawadzki et al. [80], the last decomposition stage indicates the depolymerization of chitosan chains via the deacetylation and cleavage of glycosidic linkages. Furthermore, as the decomposition temperature range meets that observed in the zein fiber curve, it can also be attributed to the protein breakdown and peptide bond cleavage [36].

The TG curves indicate a change in the material’s thermal stability by adding the chitosan layer, which was slightly improved by adding JPE. The residual mass loss of the samples was 41.5, 34.7, and 35.3% for the 30% zein fiber, 30% chitosan/zein fiber, and chitosan/zein fiber 30% + 3% JPE, respectively. A similar result was observed by Avila et al. [30], which reported that the thermal stability of zein fibers was proportionally improved by adding JPE at different concentrations in the zein matrix. Based on this, it is possible to state that there was efficient incorporation of JPE into the zein fibers, resulting in an active bilayer chitosan/zein film with good thermal stability. These results help to comprehend the material’s behavior, especially when adding the natural extract. Furthermore, thermal stability is an important factor in food packaging that requires a material resistant to processes such as hot filling and humid heat sterilization [61].

### 3.6. FTIR Analysis

**Figure 6:**
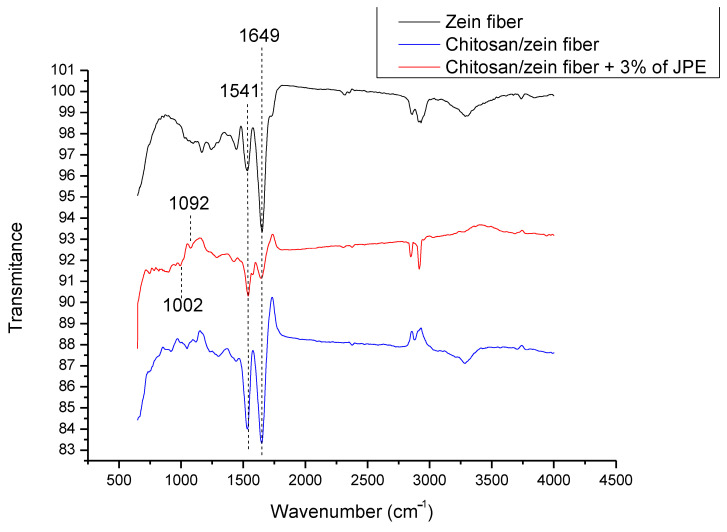
FTIR spectra of zein fiber, chitosan/zein fiber, and chitosan/zein fiber + 3% of JPE.

FTIR analysis of the zein fiber and the bilayer films was performed, and the spectra are shown in Figure 6.

The 1541 and 1649 cm^−1^ bands in all of the samples can be attributed to the C=O stretching vibration of amide I and N–H bending and the C–N stretching vibration of amide II, which are both characteristics of chitosan and zein biopolymers. In addition, some authors have reported similarities in these spectra, such as Kimna et al. [81], who developed a zein-based multilayer membrane, and Zhang et al. [56], who developed a chitosan/zein bilayer film. 

The FTIR spectrum of chitosan/zein containing JPE showed new and subtle signals. Bands at 1002 and 1092 cm^−1^ corresponding to the aromatic ring C-H deformation and stretching of pyran rings related to the phenolic compounds present in JPE were found. In line with this, when analyzing anthocyanin extract, Pereira Jr. et al. [82] were able to confirm the presence of the two peaks mentioned above. 

It is also possible to observe a broadening in the band at 1649 cm^−1^. This trend indicates the interaction between the extract and biopolymer, especially due to the hydrogen bonding between the OH groups from the phenolic compounds and the C=O of amide I. According to Wang et al. [83], the polyphenols present in the plant matrix can form hydrogen and covalent bonds, occupying functional groups, such as C=O and N-H, from biopolymers. Therefore, the presence of JPE in the biopolymer matrix was evidenced, as well as the interaction between them. In addition, electrospinning proved a good technique for active film formation since the bioactive compounds were preserved.

### 3.7. Microbial Inhibition Potential (MIP)

**Figure 7:**
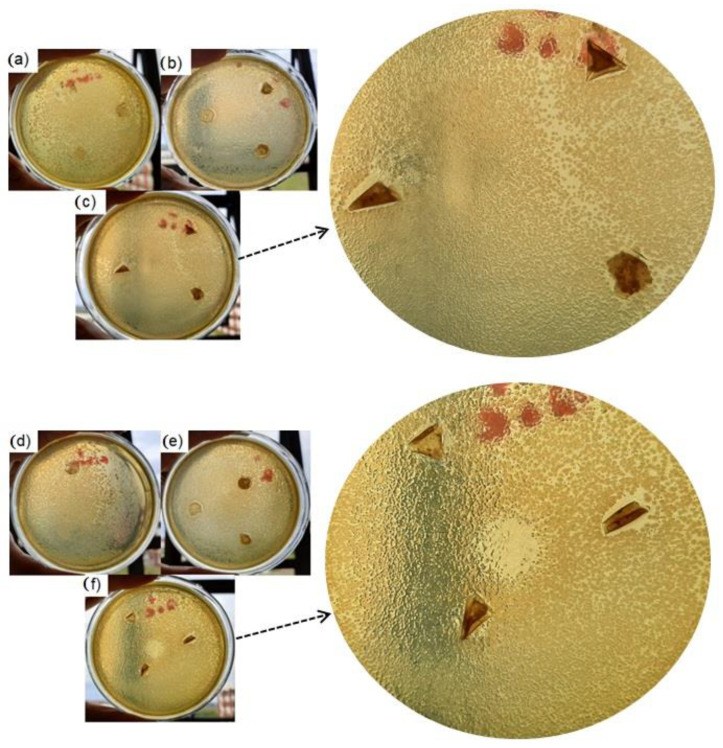
Microbial inhibition potential against *E. coli* for zein fiber (**a**), chitosan/zein fiber, (**b**) and chitosan/zein fiber + 3% of JPE (**c**) and against *S. aureus* for zein fiber (**d**), chitosan/zein fiber, (**e**) and chitosan/zein fiber + 3% of JPE (**f**).

The zein fiber and the bilayer films were also evaluated according to their microbial inhibition potential against *E. coli* and *S. aureus.* Figure 7 shows the visual inhibition test and the results, expressed as inhibition halos, which are presented in Table 5.

The bilayer film containing JPE showed an inhibition zone against the two microorganisms evaluated: *E. coli* and *S. aureus*. The extract’s potential for microbial inhibition can be attributed to the nonadditive membranes that did not present inhibition zones. Furthermore, the literature reports that the composition of this extract, which is rich in polyphenols, promotes effects such as antimicrobial action [31]. The present study’s HPLC results reinforce this statement since the individual phenolic compounds and anthocyanins identified in JPE are known for their antioxidant and antimicrobial actions.

The results were generally competitive compared to those reported in the literature. For example, Bhullar et al. [84] found an inhibition zone of 8.1 mm against *S. aureus* and *E. coli* for poly (ε-caprolactone) (PCL) films, 12.1 mm against *S. aureus,* and 8.1 mm against *E. coli* for PCL films with added natural extract from Rosemary. Hanani et al. [85] developed gelatin/polyethylene bilayer films containing different fruit peels and obtained an inhibition zone against *E. coli* of 8.00 ± 1.41 mm using pomegranate fruit peel at 7% (*w*/*v*) concentration. Besides that, the microbial inhibition potential was more pronounced against *S. aureus* than the *E. coli* microorganism. This fact can be attributed to the structure of Gram-negative bacteria since these microorganisms have a resistant and protective peptidoglycan layer [86,87,88]. Therefore, the inhibition test showed important and promising results for future applications of the bilayer material. 

## 4. Main Findings and Future Perspectives

(I) Jaboticaba peels constitute so-called agroindustrial residues. However, the extracts obtained in this study revealed their high potential for use as a natural additive. Therefore, this initiative adds value to waste and contributes to solid waste management. 

(II) The development of biopolymeric materials, especially for food packaging applications, is an essential alternative to minimize the harmful effects on nature. However, biopolymeric materials generally present poor barrier properties that limit their use. In this regard, using a bilayer structure is a noteworthy strategy to improve the fundamental parameters required for food packaging.

(III) Electrospun fibers can be combined with bilayer structures forming a material with important characteristics, such as high surface area, good malleability, and lightness. In addition, when associated with an additive, it can promote an effect known as controlled release.

(IV) Regarding the Characterization of the bilayer film, the results show that adding a second layer (chitosan layer) improved the properties of the zein fiber. This result agrees with the claim that electrospun fibers need a substrate for successful application. In addition, the purpose of the bilayer structure was also confirmed as a viable alternative for the production of biopolymer materials.

(V) The results presented in the present work are an important step towards developing environmentally friendly materials with promising applications. However, further testing is needed to confirm the controlled release property, and in vivo testing is needed to better assess the antimicrobial property of the material for food packaging.

## 5. Conclusions

JP was shown to be an important source of phenolic compounds; this reinforces the idea of recovering bioactive compounds from agroindustrial wastes for application as natural additives. More specifically, the extract showed high values of phenolic compounds, total anthocyanin, and antioxidant activity. In addition, the phenolic profile revealed that among the compounds identified, the cyanidin-3-glucoside constituted the majority. Thus, the phytochemical characteristics of JPE justified its use as a natural additive in bilayer films, which were obtained by casting and electrospinning techniques. Adding a second layer composed of chitosan resulted in an increase in thickness compared to the zein fiber layer and improvements in important parameters, such as solubility in water and water vapor permeability. Besides that, the incorporation of JPE in the polymeric matrix did not significantly affect these parameters concerning the bilayer film without JPE.

On the other hand, the addition of JPE affected the morphology of the zein fibers since the zein fiber decreased when deposited into a chitosan film and continuously decreased in the bilayer chitosan/zein fiber + 3% of JPE. This trend is possibly related to the AV and EC of zein solutions which increased with the addition of JPE. In the same line, the roughness of the zein fibers also reduced with the addition of JPE, resulting in a less porous material. Furthermore, thermal stability was also improved with the addition of JPE, and FTIR analysis helped to confirm its presence in the polymer matrix. Finally, the active potential of the bilayer film was proved through the antimicrobial analysis that showed inhibition halos against *E. coli* and *S. aureus*. The results of this study suggest that using a bilayer system is an interesting option to improve the barrier properties of biopolymer materials, making them more competitive against synthetic packaging materials. In addition, a natural extract as an additive capable of conferring unique properties to the final material, such as antioxidant and antimicrobial potential, was also confirmed. Thus, the material developed in the present study is promising for use as active food packaging.

## Figures and Tables

**Table 1:** Identified phenolic compounds in JPE.

Compounds	Concentration (mg 100 g^−1^)
Gallic acid	177.02 ± 4.38
Caffeic acid	87.15 ± 3.11
*p*-Coumaric acid	203.09 ± 4.96
*Trans*-Ferulic acid	91.75 ± 3.71
Kaempferol	46.26 ± 0.68
Cyanidin-3-glucoside	1857.81 ± 10.98

Mean ± mean deviation (n = 2).

**Table 2:** Apparent viscosity and electrical conductivity of zein solutions.

	Zein	Zein + 3% JPE
AV (Pa s)	0.472 ± 0.002 ^a^	0.776 ± 0.002 ^b^
EC (μs cm^−1^)	94.55 ± 0.15 ^a^	277.5 ± 0.5 ^b^

Mean ± mean deviation (n = 2). Different letters in the same column indicate significant differences between the samples for the *t*-test (*p* < 0.05).

**Table 3:** Characterization of the zein fiber and bilayer films according to thickness, solubility in water, and water vapor permeability.

	Zein Fiber	Chitosan/Zein Fiber	Chitosan/Zein + 3% of JPE
Thickness (mm)	0.19 ± 0.03 ^b^	0.51 ± 0.04 ^a^	0.50 ± 0.05 ^a^
Solubility in water (%)	36.50 ± 4.68 ^a^	12.96 ± 0.92 ^b^	27.38 ± 0.05 ^a,b^
Water vapor permeability (g m^−1^ Pa^−1^ s^−1^)	4.48 × 10^−9^ ± 1.21 × 10^−10 a^	1.6 × 10^−10^ ± 7.38 × 10^−12 b^	1.58 × 10^−10^ ± 6.92 × 10^−12 b^

Mean ± mean deviation (n = 10 for thickness, n = 3 for solubility in water, and n = 3 for WVP). Different letters in the same line indicate significant differences between samples in the Tukey test (*p* < 0.05).

**Table 4:** Roughness and mean diameter of zein fiber, chitosan/zein fiber, and chitosan/zein fiber + 3% of JPE.

	Zein Fiber	Chitosan/Zein Fiber	Chitosan/Zein + 3% of JPE
Roughness (μm)	0.19 ± 0.03 ^b^	0.51 ± 0.04 ^a^	0.50 ± 0.05 ^a^
Mean diameter (μm)	36.50 ± 4.68 ^a^	12.96 ± 0.92 ^b^	27.38 ± 0.05 ^a,b^

Mean ± mean deviation (n = 2 for roughness, n = 6 for mean diameter). Different letters in the same column indicate significant differences between the samples for the *t*-test (*p* < 0.05).

**Table 5:** The inhibition of halos (mm) against *E. coli* and *S. aureus* of the zein fiber and the bilayer films.

	Zein Fiber	Chitosan/Zein	Chitosan/Zein + 3% of JPE
*E. coli*	-	-	8.77 ± 0.31
*S. aureus*	-	-	9.32 ± 0.21

Mean ± mean deviation (n = 3).

## Data Availability

Not applicable.
